# Stability Improvement of Solution-Processed Metal Oxide Thin-Film Transistors Using Fluorine-Doped Zirconium Oxide Dielectric

**DOI:** 10.3390/ma18091980

**Published:** 2025-04-27

**Authors:** Haoxuan Xu, Bo Deng, Xinan Zhang

**Affiliations:** Shenzhen Key Laboratory of Ultraintense Laser and Advanced Material Technology, College of Engineering Physics, Shenzhen Technology University, Shenzhen 518118, China

**Keywords:** fluorine-doped, ZrO_2_ dielectric, thin-film transistor, electrical performance

## Abstract

Solution-processed metal oxide dielectrics often result in unstable thin-film transistor (TFT) performance, hindering the development of next-generation metal oxide electronics. In this study, we prepared fluorine (F)-doped zirconium oxide (ZrO_2_) dielectric layers using a chemical solution method to construct TFTs. The characterization by X-ray photoelectron spectroscopy (XPS) revealed that appropriate fluoride doping significantly reduces oxygen vacancies and the concentration of hydroxyl groups, thereby suppressing polarization processes. Subsequently, the electrical properties of Al/F:ZrO_2_/n^++^Si capacitors were evaluated, demonstrating that the optimized 10% F:ZrO_2_ dielectric exhibits a low leakage current density and stable capacitance across a wide frequency range. Indium zinc oxide (IZO) TFTs incorporating 10% F:ZrO_2_ dielectric layers were then fabricated. These devices displayed reliable electrical characteristics, including high mobility over a broad frequency range, reduced dual-sweep hysteresis, and excellent stability under positive-bias stress (PBS) after three months of aging. These findings indicate that the use of the fluorine-doped ZrO_2_ dielectric is a versatile strategy for achieving high-performance metal oxide thin-film electronics.

## 1. Introduction

Metal oxide thin-film transistors (TFTs) have garnered significant attention for their potential applications in advanced electronics owing to their high carrier mobility, large-area uniformity, high optical transparency, and electrical stability [1,2,3,4]. Generally, a high capacitance in thin-film transistors (TFTs) markedly augments the capacitive coupling between the gate and the active layer. Consequently, considerable efforts have been dedicated to the development of high-k dielectric thin films with the aim of reducing power consumption, thus facilitating the realization of mobile and portable applications [5,6]. Among the various deposition techniques, such as radio frequency sputtering, chemical vapor deposition (CVD), atomic layer deposition (ALD), inkjet printing, and spin-coating, solution processing has arisen as a valuable approach owing to its simplicity, low cost, facile control of chemical stoichiometry, and scalability for mass production. Therefore, solution-processed high-k oxide dielectrics, including zirconium oxide (ZrO_2_), hafnium oxide (HfO_2_), yttrium oxide (Y_2_O_3_), and aluminum oxide (Al_2_O_3_), have attracted substantial research interest for low-voltage thin-film devices [7,8,9]. Among these, ZrO_2_ emerges as a promising candidate owing to its high dielectric constant and exceptional thermodynamic stability. ZrO_2_, in particular, has a wide bandgap, which plays an important role in optoelectronic devices [10]. However, ZrO_2_ inherently exhibits polarization mechanisms, leading to imperfect insulating properties. This results in a strong capacitance-frequency dependence and high dielectric losses, causing irreversible current hysteresis in oxide TFTs [11,12]. To address these issues, anionic fluorine (F) doping has been employed to modify metal oxide semiconductor materials and dielectrics. Fluorine ions, with a similar radius to oxygen ions, not only minimize lattice distortions but also enable the substitution of oxygen atoms, creating free electrons or occupying oxygen vacancy sites to eliminate electron traps. For instance, Sil et al. fabricated organic TFTs with reduced I-V hysteresis and improved bias stress stability using the fluorine-doped ZrO_2_ dielectric [11]. Additionally, Zhuang et al. achieved a stable frequency capacitance by doping the Al_2_O_3_ dielectric with fluorine, and their devices exhibited reliable electrical characteristics and minimal hysteresis [13]. It is also crucial to use advanced characterization techniques such as electron holographic tomography to study the internal potential of these devices in order to investigate their unique properties [14]. In this study, a solution-processed method was employed to achieve a fluorine-doped ZrO_2_ dielectric with stable capacitance across a broad frequency range. The morphology, chemical binding states, and electrical performance metrics of the ZrO_2_ thin films were systematically investigated. Ultimately, solution-processed indium zinc oxide (IZO) TFTs with enhanced stability were integrated using the optimized ZrO_2_ dielectrics.

## 2. Materials and Methods

### 2.1. Preparation of the Precursor

All chemicals were procured from Aladdin and utilized without further purification. In order to prepare the IZO channel layer, an IZO (In:Zn = 1:0.2) precursor solution was prepared by dissolving appropriate amounts of In(NO_3_)_3_·xH_2_O (99.999%) and Zn(NO_3_)_2_·xH_2_O (99.999%) in 2-methoxyethanol to produce a 0.05 M solution. For the F:ZrO_2_ solution, 0.3 M ZrO(NO_3_)_2_·xH_2_O (99.99%) and varying molar concentrations (0%, 5%, 10%, and 15%) of HF were combined with 2-methoxyethanol. All precursor solutions were stirred overnight at 25 °C and subsequently filtered through a 0.2 µm polytetrafluoroethylene membrane filter prior to spin coating.

### 2.2. Device Fabrication

Bottom-gate-structured TFTs were fabricated on an n^++^ silicon wafer (100) substrate. Initially, the substrate underwent ultrasonic cleaning in acetone, ethanol, and deionized water sequentially for 10 min each, followed by O_2_ plasma treatment. The ZrO_2_ dielectric was deposited onto the substrate via spin coating at 3000 rpm for 30 s and then annealed at 200 °C for 20 min on a hotplate. Upon heating, the hydrated nitrate salts underwent thermal decomposition, followed by the breakdown of nitrate ions (NO_3_⁻) into nitrogen oxides (NOx) while releasing water vapor. Simultaneously, indium and zinc ions react to form a mixed metal oxide. This process was repeated three times to attain the desired thickness. The samples were then annealed at 500 °C for 1 h and subjected to ultraviolet treatment for 30 min. Next, thermally evaporated circular aluminum electrodes were deposited onto the ZrO_2_ dielectric layer via a shadow mask to form Al/ZrO_2_/n^++^Si metal–insulator–semiconductor (MIS) structures. For the IZO/ZrO_2_ TFT devices, the IZO precursor was spin-coated onto the ZrO_2_ substrate at 3000 revolutions per minute (rpm) for 30 s and subsequently annealed at 300 °C for 20 min. Finally, the aluminum source and drain top electrodes were deposited by thermal evaporation through a shadow mask to define a channel width of 1000 µm and a channel length of 100 µm.

### 2.3. Characterization

The surface morphology and roughness were characterized using a Veeco Dimension Icon atomic force microscope (AFM) in tapping mode. An X-ray photoelectron spectroscopy (XPS) analysis was conducted using an Escalab 250 Xi spectrometer (Thermo Fisher Scientific, Pardubice, Czech Republic). Film thickness was measured using a M2000U ellipsometer (J. A. Woollam, Lincoln, NE, USA) and fit with a Cauchy model. The dielectric capacitance of the MIS capacitors as a function of frequency, ranging from 20 Hz to 1 MHz, was evaluated using an Agilent E4980A precision LCR meter (Santa Rosa, CA, USA). The leakage current–voltage characteristics of the MIS capacitors and TFTs were measured using a Keithley 2450 semiconductor parameter analyzer (Cleveland, OH, USA).

## 3. Results

**Figure 1:**
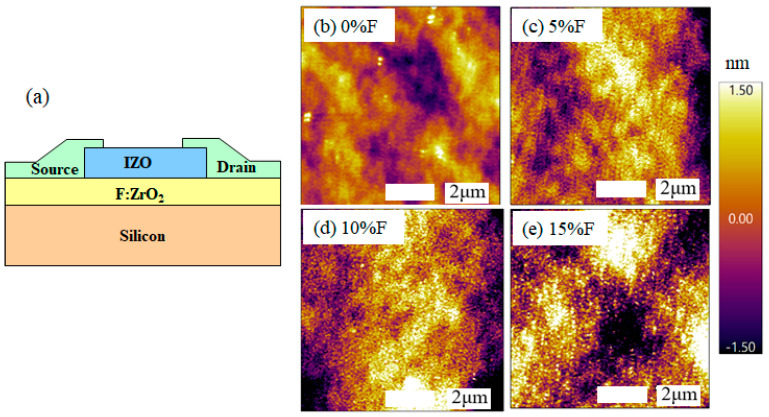
(**a**) Schematic structure of the TFT. Atomic force microscopy (AFM) images of F:ZrO_2_ thin films with varying fluorine doping concentrations: (**b**) 0%, (**c**) 5%, (**d**) 10%, and (**e**) 15%.

Figure 1 presents the schematic structure of the TFT, and the AFM images of ZrO_2_ films doped with different concentrations of fluorine. Figure 1b–e correspond to 0%, 5%, 10%, and 15% of F:ZrO_2_ films, respectively. These films exhibit dense and continuous surface morphologies with scattered small particles. The root mean square (RMS) roughness values are 0.473 nm, 0.488 nm, 0.539 nm, and 0.753 nm, respectively. It was observed that the surface roughness increases with the increasing doping concentration. In general, however, these F:ZrO_2_ films are dense, uniform, and quite smooth, making them suitable for use as insulating layers in TFTs [15,16].

**Figure 2:**
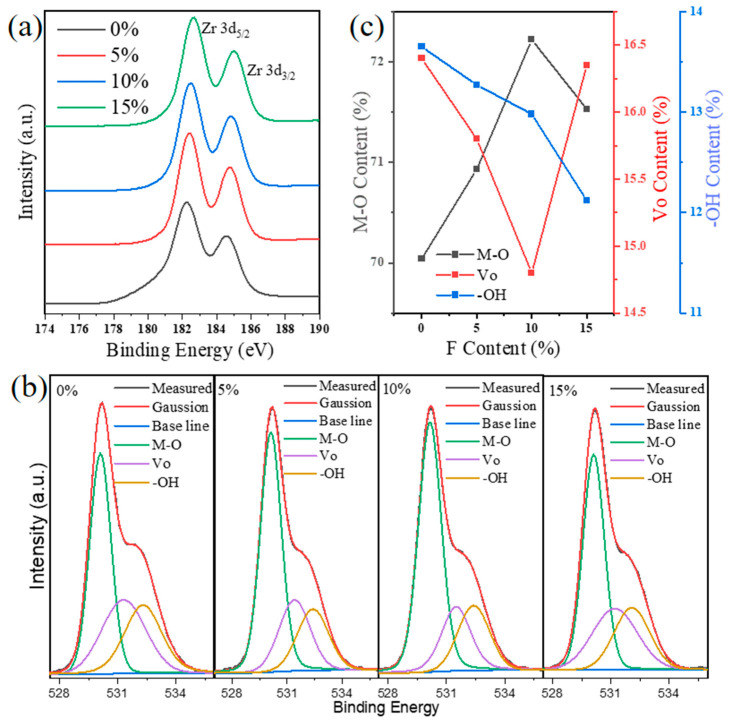
XPS spectra of F:ZrO_2_ thin films with different F doping concentrations. (**a**) Zr 3d spectra; (**b**) best-fit results for O1s peaks. (**c**) Atomic percentages of M-O, VO, and -OH bonds as a function of the F content.

Figure 2 illustrates the XPS spectra of ZrO_2_ films doped with varying F concentrations. In Figure 2a, the spin–orbit doublet peaks of Zr 3d (3d^5/2^ and 3d^3/2^) were observed with no additional impurity peaks. The peak centers were 182.20 and 184.60 eV, 182.40 and 184.80 eV, 182.60 and 185.00 eV, and 182.50 and 184.80 eV, respectively. The separation of 2.4 eV between the Zr 3d spin–orbit doublets suggests the presence of ionic bonding between Zr^4+^ and O^2−^ in the ZrO_2_ films. Compared to the ZrO_2_ sample, the Zr 3d peak shifted 0.02 eV, 0.04 eV, and 0.03 eV towards higher binding energy for the 5%, 10%, and 15% F:ZrO_2_ films, respectively. This shift may be attributed to a decrease in the coordination number of metal ions in the F:ZrO_2_ films [17,18].

In Figure 2b, the O1s peaks of F:ZrO_2_ films are presented. Through Gaussian peak fitting, the O1s peak is resolved into three sub-peaks corresponding to different oxygen environments, centered at 530.02 eV, 531.37 eV, and 532.41 eV, respectively. The primary lattice peak of O1s at 530.02 eV is attributed to lattice oxygen (M-O) in metal oxides, while the peaks at 531.37 eV and 532.41 eV are associated with oxygen vacancies (V_O_) and hydroxyl groups (-OH) in metal hydroxides, respectively [19]. By analyzing the ratio of lattice oxygen, V_O_, and -OH to the total area of the O1s XPS spectrum, the relative contents could be determined, as shown in Figure 2c. This method is widely used to evaluate the quality of oxide thin films [20,21]. Through data comparison, it is evident that as the F doping concentration increases from 0% to 10%, the M-O bond ratio rises from 70.04% to 72.22%, while the Vo content decreases from 16.40% to 15.80%, and the -OH content drops from 13.65% to 12.12%. After F doping, the reduction in the Vo and -OH contents can be attributed to F occupying some of the oxygen vacancies, thereby decreasing the oxygen vacancy density [22,23]. Due to the comparable ionic radii between F⁻ and O^2^⁻, fluorine substitution not only mitigates structural distortions in the lattice but also replaces oxygen atoms, either generating delocalized electrons or passivating vacancy-related traps for enhanced charge transport. Additionally, the high electronegativity of F ions allows them to form hydrogen bonds with -OH, passivating the trap sites induced by -OH. However, the oxygen vacancy content of the 15% F:ZrO_2_ film unexpectedly increases to 16.35%, possibly due to excessive F doping leading to an increase in defects within the ZrO_2_ film [24].

**Figure 3:**
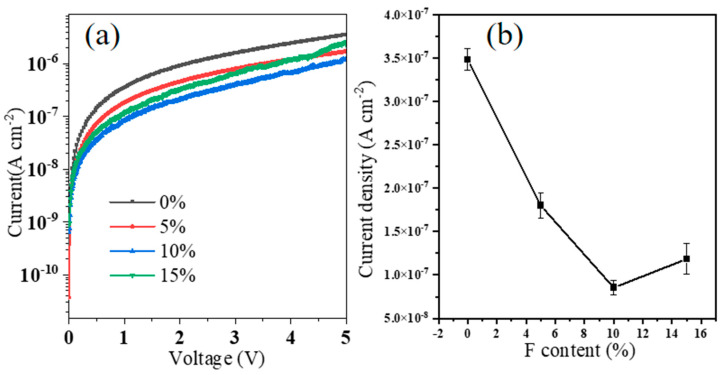
(**a**) The leakage current–voltage characteristic curves of F:ZrO_2_ films with different F concentrations. (**b**) The leakage current density as a function of F concentrations.

Figure 3a illustrates the leakage–current behavior of F:ZrO_2_ films with varying F doping concentrations. The leakage–current density of ZrO_2_ was measured to be 3.5 × 10^−7^ A/cm^2^ at 2 V. This density gradually decreased to 1.8 × 10^−7^ A/cm^2^ for 5% F:ZrO_2_ and 8.4 × 10^−8^ A/cm^2^ for 10% F:ZrO_2_, likely due to F ions occupying oxygen vacancies, reducing defects, and enhancing the film’s density and insulation properties. This observation is consistent with the reduced oxygen vacancies observed in the XPS results. Conversely, the leakage current density for 15% F:ZrO_2_ increased to 1.2 × 10^−7^ A/cm^2^, potentially because the doped F replaced oxygen atoms, generating free electrons and increasing the carrier concentration [25]. The relationship between the leakage current density of F:ZrO_2_ at 2 V and the F doping concentration is depicted in Figure 2b. The highest standard deviation of the entire leakage current density was about 12.5%, which proves that the device preparation has good repeatability. Furthermore, the graph indicates that all films exhibit a high breakdown electric field, effectively preventing the insulation layer from being compromised by the gate field.

**Figure 4:**
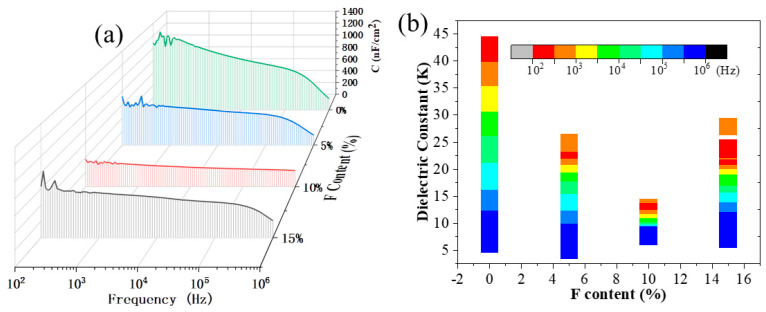
(**a**) Capacitance–frequency plots in the range of 100 Hz to 1 MHz for F:ZrO_2_ films with different F concentrations. (**b**) The corresponding dielectric constants are shown at the indicated frequencies.

Next, capacitance–frequency curves of the Al/F:ZrO_2_/n^++^Si MIS capacitors were measured in the range of 100 Hz to 1 MHz using a bias voltage of 1 V, as illustrated in Figure 4a. The capacitance per unit area (Ci) of all F:ZrO_2_ films was observed to gradually decrease with increasing frequency. This phenomenon can be attributed to capacitive dispersion in the high-k material, which arises from electron polarization, ion polarization, and interfacial polarization [6,26]. The capacitance of the undoped ZrO_2_ film exhibited the greatest variation, ranging from 1023 nF/cm^2^ at 100 Hz to 206 nF/cm^2^ at 1 MHz. Notably, F doping consistently improved the stability of capacitance compared to the undoped ZrO_2_. This suggests a denser formation of M-O bonds and a lower density of defects such as oxygen vacancies (V_O_) and hydroxyl groups (-OH) in the thin film. On the one hand, this improvement may be due to the filling of oxygen vacancies in ZrO_2_ films with F ions, thereby reducing defect density. On the other hand, it could be attributed to the formation of hydrogen bonds between the doped F and hydroxyl groups, which passivate the trap sites induced by -OH, significantly reducing the number of traps in the insulating layer and, thus, minimizing capacitive dispersion [27,28]. Among the samples, the 10% F:ZrO_2_ film demonstrated the most stable capacitance over a broad frequency range, from 358.6 nF/cm^2^ at 100 Hz to 229.2 nF/cm^2^ at 1 MHz, which is crucial for reliable device operation at high frequencies [29,30]. However, when the F content is increased to 15%, the capacitive dispersion worsens, likely due to the introduction of additional defects caused by excessive F doping. Additionally, the thicknesses of the F:ZrO_2_ films, as determined by spectroscopic ellipsometry, were approximately 32.5 nm. The frequency dependence of the k values was calculated by the equation k = Cd/ε_0_S, where ε_0_ is the permittivity of a vacuum, C is the capacitance of the dielectric layer obtained from the capacitor test, S is the overlapping area of the two plates, and d is the thickness of the dielectric layer. The results are presented in Figure 4b. It is evident that the 10% F:ZrO_2_ film exhibits relatively stable dielectric constants.

**Figure 5:**
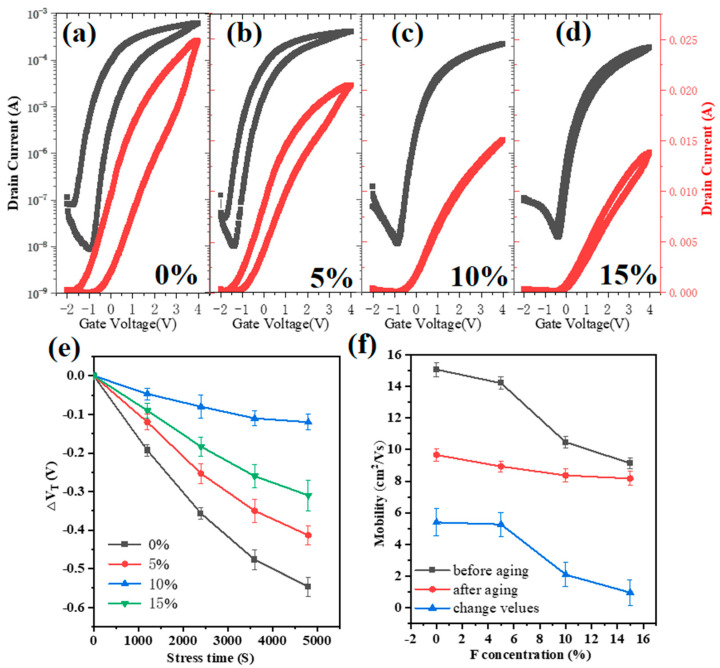
(**a**–**d**) Transfer curves of IZO/F:ZrO_2_ TFTs with different F concentrations. (**e**) The threshold voltage shift of ΔVth after a 4800 s gate bias was applied. (**f**) The fluctuations in average mobility of these devices after three months.

To evaluate the suitability of F:ZrO_2_ films as gate dielectrics in oxide thin-film transistors, we fabricated bottom-gate IZO TFTs incorporating 0%, 5%, 10%, and 15% of F-doped ZrO_2_ dielectrics. Representative transfer curves of IZO/F:ZrO_2_ TFTs with VDS = 4 V are shown in Figure 5a–d. All samples exhibited anticlockwise hysteresis, indicating that the gate capacitance varies during continuous VGS sweeps from −2 V to 4 V and back to −2 V. Due to the higher capacitance at low frequencies under bias, the prolonged application of a larger VGS gradually increased the capacitance, leading to consistently higher capacitance during the backward sweep. Consequently, the IDS value was always greater during the backward sweep compared to the forward sweep at a given V_GS_, resulting in anticlockwise hysteresis [31]. For devices with an F-doped ZrO_2_ dielectric, improved transfer curves with reduced hysteresis were observed. Specifically, the 5% F:ZrO_2_-based TFT showed a reduced anticlockwise hysteresis of 0.86 V compared to the undoped ZrO_2_-based TFTs, which exhibited a hysteresis of 1.16 V. For 10% F:ZrO_2_-based TFTs, the hysteresis was only 0.12 V, suggesting that appropriate F doping significantly reduces the I-V hysteresis. This effect is likely due to F ions occupying oxygen vacancy (V_O_) sites in the film, thereby reducing dielectric bulk defects, enhancing film density, and minimizing the interface defects between IZO and F:ZrO_2_ [32]. However, for 15% F:ZrO_2_-based TFTs, the anticlockwise hysteresis increased to 0.26 V, possibly due to an increase in interfacial defects caused by excessive F doping. Positive gate bias stress (PBS) tests on the TFTs were conducted at V_DS_ = 1 V for 4800 s, with the results shown in Figure 5e. The 10% F:ZrO_2_-based TFTs exhibited the smallest ΔV_th_ during the gate bias process. These findings demonstrate that F doping is an effective method to enhance the bias stress stability of devices.

Given that the gate dielectric capacitance is frequency-dependent, we systematically evaluated the frequency dependence of carrier mobility. All calculations were based on the forward sweeping curve when hysteresis was observed in the devices. The results clearly indicate that undoped ZrO_2_-based TFTs exhibit significant mobility variations, ranging from 26.8 cm^2^V^−1^s^−1^ at 1 MHz to 3.5 cm^2^V^−1^s^−1^ at 100 Hz. Upon the fluorine doping of the gate dielectric, the mobility range significantly narrows. Notably, for 10% F:ZrO_2_ TFTs, the calculated mobility remained within a narrow range of 13.8 cm^2^V^−1^s^−1^ at 1 MHz to 7.3 cm^2^V^−1^s^−1^ at 100 Hz, with an average value of 10.5 cm^2^V^−1^s^−1^.

The electrical performance of IZO/F:ZrO_2_ TFTs with varying F concentrations was also assessed after three months of aging in ambient air without surface passivation. Figure 5f illustrates the fluctuations in average mobility. Specifically, the average mobility values changed as follows: from 15.1 to 9.6 cm^2^V^−1^s^−1^ for undoped IZO/ZrO_2_ TFTs, from 14.2 to 8.9 cm^2^V^−1^s^−1^ for 5% F:ZrO_2_ TFTs, from 10.5 to 8.3 cm^2^V^−1^s^−1^ for 10% F:ZrO_2_ TFTs, and from 9.1 to 8.2 cm^2^V^−1^s^−1^ for 15% F:ZrO_2_ TFTs over the three-month period. The corresponding changes in mobility gradually decreased with the increasing F concentration. These findings suggest that F ions play a crucial role in mitigating device aging [33,34].

## 4. Conclusions

In summary, we successfully fabricated solution-processed F:ZrO_2_ thin films and systematically investigated their surface morphology and oxygen defect states. It was confirmed that F ions can occupy oxygen vacancies appropriately, reducing the density of oxygen vacancies while suppressing polarization processes. The leakage–current (I–V) and capacitance–frequency (C–f) characteristics of Al/F:ZrO_2_/n^++^Si capacitors were evaluated, revealing that the F:ZrO_2_ dielectric exhibits low leakage current density and stable capacitance over a wide frequency range. Subsequently, metal oxide TFTs with IZO as the channel layer and F:ZrO_2_ as the dielectric were fabricated. The optimized 10% F:ZrO_2_-based TFTs exhibited reliable electrical characteristics, including high mobility across a broad frequency range from 100 Hz to 1 MHz, reduced dual-sweep hysteresis, and excellent stability under prolonged bias stress and after three months of aging. These characteristics can be attributed to the similarity in radius between F ions and O ions, which notably mitigates lattice distortion and eliminates electron traps. These results suggest that fluorine-doped ZrO_2_ is a promising dielectric for applications in metal oxide thin-film electronics.

## Data Availability

The original contributions presented in this study are included in the article. Further inquiries can be directed to the corresponding author.
